# Does the Rechallenge with Another CDK 4/6 Inhibitor in Breast Cancer Work? A Case Report and Literature Review

**DOI:** 10.3390/medicina59040696

**Published:** 2023-04-01

**Authors:** Ioana-Miruna Stanciu, Cristina Florina Pirlog, Andrei-Wilhelm Anghel, Andreea Ioana Parosanu, Cristina Mihaela Olaru, Cristina Orlov-Slavu, Ion Cristian Iaciu, Ana Maria Popa, Radu Constantin Vrabie, Cornelia Nitipir

**Affiliations:** 1 Department of Oncology, “Carol Davila” University of Medicine and Pharmacy, 020021 Bucharest, Romania; ioana-miruna.stanciu@drd.umfcd.ro (I.-M.S.);; 2 Department of Oncology, Elias University Emergency Hospital, 011461 Bucharest, Romania; 3 Department of Radiotherapy, Elias University Emergency Hospital, 011461 Bucharest, Romania

**Keywords:** CDK4/6 inhibitors, CDK4/6 rechallenge, CDK4/6 toxicity, ribociclib, abemaciclib

## Abstract

Cyclin-dependent kinase 4 and 6 (CDK4/6) inhibitors and endocrine therapy are the gold standards for systemic therapy for patients with hormone-positive (HR+)/human epidermal growth factor receptor-2-negative (HER2-) metastatic breast cancer. Following progression, no prospective randomized data exist to help guide second-line treatment. Moreover, there is a scarcity of data on rechallenge treatment strategies with another CDK4/6 inhibitor after prior limiting toxicity. We report a real-world experience of rechallenging with abemaciclib after the prior reaction of grade 4 liver toxicity to ribociclib, with high transaminases values of more than 27 times the upper limit of normal (ULN) and unexpected grade 3 neutropenia and diarrhea after a few months of abemaciclib. After two years of treatment, the patient had stable oncological disease, with normal complete blood count, hepatic enzymes, and a very good performance status. We believe that our clinical case, along with others gathered from all around the world, will help with the consolidation of an unmet clinical need to readjust the treatment after experiencing toxicity to CDK4/6 inhibitors.

## 1. Introduction

Breast cancer has the highest incidence rate and is the second leading cause of cancer-related deaths among all cancers for both sexes and all ages worldwide, according to GLOBOCAN 2020. Hormone receptor-positive (HR+)/human epidermal growth factor receptor-2-negative (HER2-) metastatic breast cancers constitute the most common subset of breast cancer, representing about three-fourths of these cancers [1]. The current standard of care for the first-line treatment of metastatic HR+/HER2- breast cancer is an aromatase inhibitor (AI) in combination with a cyclin-dependent kinase (CDK) 4/6 inhibitor. Now, three CDK4/6 inhibitors have been approved by the Food and Drug Administration (FDA) for the treatment of HR+/HER2- metastatic breast cancer: palbociclib, ribociclib, and abemaciclib. The addition of these agents to endocrine therapy has resulted in the longest improvement to date in progression-free survival (PFS) in this subtype of breast cancer [2]. It is worth noting that the three approved CDK4/6 inhibitors present distinct relative potencies for CDK4 and CDK6 inhibition, pharmacokinetics, dosing schedules, and toxicity profiles [2].

Limited data exist on rechallenge treatment strategies with CDK4/6 inhibitors after limiting toxicity. Until results from randomized studies shed light on this, empirical and real-world evidence may help make individualized decisions on CDK4/6 inhibitors rechallenge after induced toxicities.

Here, we report the case of a patient presenting grade four hepatic toxicity while receiving ribociclib that was rechallenged by switching to abemaciclib, which caused grade three diarrhea and neutropenia.

## 2. Case Report

**Figure 1:**
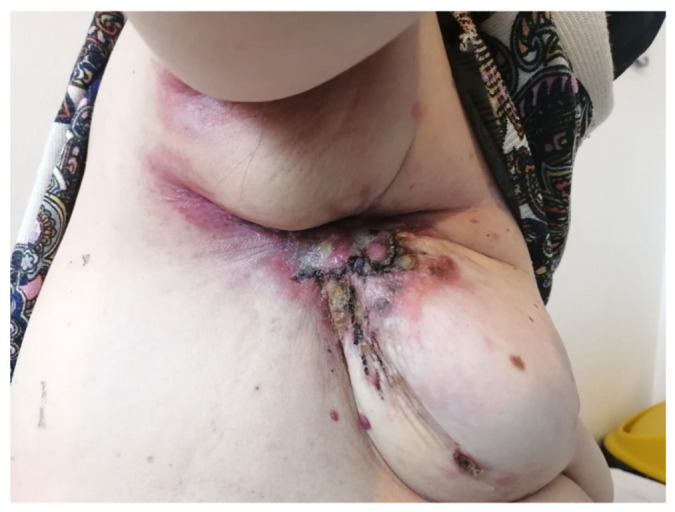
Clinical presentation of the left breast with ulceration and macroscopic nodules.

We report the case of a 67-year-old female living in a rural area who presented in our department in November 2020 complaining of dyspnea, shortness of breath on small efforts, loss of appetite, chronic fatigue, and significant weight loss (12 kg in the previous 3 months). She had no particular background, family history, or personal pathological antecedents and was a non-smoker. At the clinical examination, a massive left breast tumor, which appeared to be an inflammatory carcinoma, was revealed (Figure 1). It had a direct extension to the skin, with ulceration and macroscopic nodules. On palpation, we also detected ipsilateral fixed axillary lymph nodes. Based on these characteristics, the tumor was categorized as cT4d cN2 inflammatory breast cancer. Pulmonary auscultation and percussion revealed basal dullness and diminished pulmonary rales on the left hemithorax. On performing abdominal palpation, the liver appeared to be enlarged in dimensions. The clinical examination revealed no other findings.

Because of the patient’s clinical condition and the possibility of quick access to imaging examination, the patient underwent thorax-abdomen-pelvis computed tomography. The detailed report revealed left breast tumor formation that associated retractile radiation bands on the integument and the nipple region and came into contact with the plane of the pectoral muscles. In addition, the report showed axillary adenopathies with malignant characteristics and prepectoral tumor satellite nodules, confluent paracardiac nodular lesions on the left side (masked by massive pleural effusion of the left side) and an enlarged liver with hypoabsorbing areas with a diffused appearance and a tendency to confluence, which required magnetic resonance imaging (MRI) for better characterization.

Evacuating and exploratory thoracentesis was performed without incidents, with the evacuation of 1500 mL of serocitrine liquid. The liquid was immediately sent to the laboratory for analysis, which was concluded to be a malignant smear.

On the same day, the patient also underwent a breast biopsy with an immunohistochemistry test, which concluded that it was a poorly differentiated NST (no specific type) invasive ductal carcinoma, with perineural and lymphovascular invasion present, with immunophenotype of ER = 60%, PgR = 10%, HER2−negative, and ki67 = 80%.

**Figure 2:**
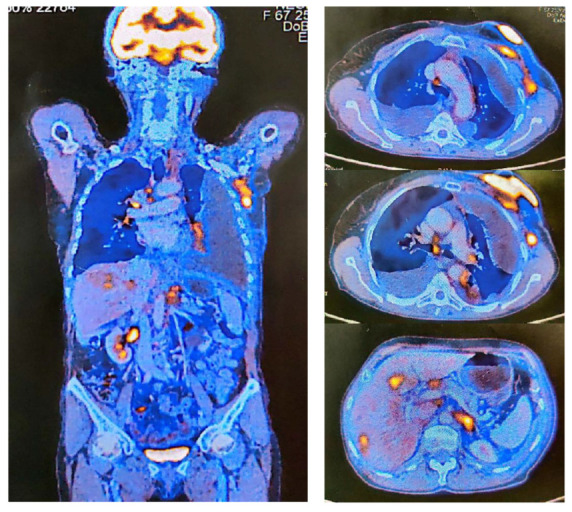
PET-CT from December 2020 with metabolically active lesions.

The patient was a candidate for positron emission tomography (PET-CT), which was done in December 2020 (Figure 2). This revealed a left breast tumor of 63/30 mm, with increased metabolic activity (SUV = 17.81) in the upper quadrants invading the integument and pectoralis major; a 13/18 mm iodophil nodule located in the lower external quadrant with increased metabolic activity (SUV = 8.7); left axillary adenopathy of 33/16 mm with increased metabolic activity; metabolically active adenopathies in the mediastinum, right paratracheal, infracarinal and right pulmonary hilar; metabolically active hepatic metastases (SUV = 7.93); left adrenal metastasis; abdominal adenopathies located on the small gastric curvature, celiac, hepatic hilar, and inter-aorto caval; and oncological staging: T4 N2 M1 (HEP, LYM, and ADR).

The multidisciplinary tumor board united and discussed the treatment possibilities for this case (chemotherapy vs. targeted therapy). Owing to its characteristics, the tumor was categorized as cT4d cN2 inflammatory breast cancer. It was decided that the best starting treatment for this patient was chemotherapy, given the ulceration of the breast, the permeation nodules, the high value of ki67 (80%), and the increased risk of visceral crisis. In addition, we chose docetaxel and not paclitaxel because of its higher activity in metastatic breast cancer and its ability to induce responses in liver metastases.

The chemotherapy regimen used was 4 cycles of AC (doxorubicin 60 mg/m^2^ IV day 1 + cyclophosphamide 600 mg/m^2^ IV day 1, cycled every 21 days) with G-CSF (pegfilgrastim 6 mg IV day 1) followed by 4 cycles of taxanes (docetaxel 100 mg/m^2^ IV day 1, cycled every 21 days), according to the NCCN Guidelines version 2020 for systemic therapy—other recommended regimens. Before starting the treatment, the patient underwent a cardiological consultation and an EKG. The treatment lasted from December 2020 to May 2021.

**Figure 3:**
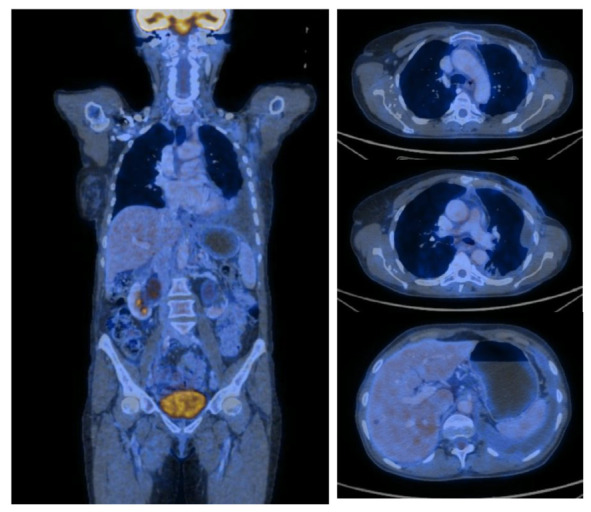
PET-CT from June 2021 with no metabolically active lesions.

At the end of the chemotherapy treatment, in June 2021, the patient underwent a second PET-CT (Figure 3) to assess the disease response. The exam revealed significant left breast dimensional regression with skin retraction and diffusely and slightly increased metabolic activity and small residual axillary lymph nodes with mild metabolic activity. In addition, multiple bone lesions of a maximum of nine mm at the level of the left iliac wing without metabolic activity (probably post-therapeutic inactivated metastases) were revealed. The exam concluded that no metabolically active PET-CT lesions were found.

The multidisciplinary oncological tumor board reunited and discussed future directions for the patient. Taking into consideration the patient’s refusal for surgical intervention, the recommendation was radiotherapy to the breast and lymph node areas, along with CDK4/6 inhibitors and AI. The treatment regimen was to give ribociclib 200 mg × 3/day orally for 3 weeks, followed by 1 week off and to give letrozol 2.5 mg/day orally as a continuous regimen.

The patient was also advised to have a dental consultation (to exclude dental infections and other pathologies) and to initiate treatment with osteoclast inhibitors (zoledronic acid 4 mg IV every 4 weeks), according to NCCN guidelines, which recommend that post-menopausal patients with node-positive tumors receive adjuvant bisphosphonate therapy for risk reduction of distant metastasis for 3–5 years. Before starting the treatment with CDK4/6 inhibitors, the patient underwent a cardiological evaluation with an EKG test and an echocardiogram. A complete blood count (CBC), including liver enzymes, was also done before starting the treatment (Table 1).

**Figure 4:**
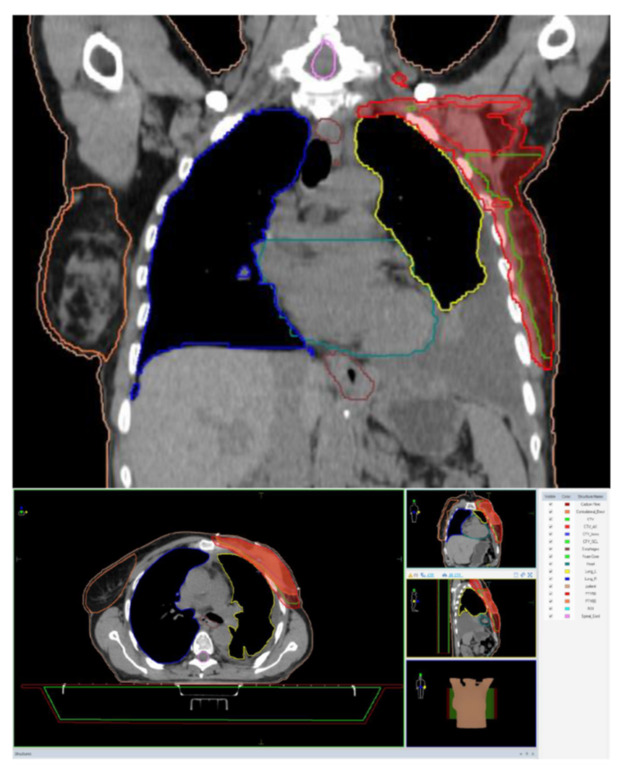
Radiation treatment planning.

During July–August 2021, the patient underwent adjuvant external radiotherapy at the level of target volumes (PTV50: left breast and locoregional lymph nodes with a total dose of 50 Gy/25 fx at 2 Gy, 5 days/week and at the tumor level with a total dose of 60 Gy/30 fx at 2 Gy, 5 days/week) using the IMRT-VMAT technique (Figure 4).

Unfortunately, after only one month of treatment with CDK4/6 inhibitors, the patient developed grade two liver toxicity with transaminases (alanine aminotransferase—ALT and aspartate aminotransferase—AST) four times the upper limit of normal (ULN). We temporarily stopped the treatment until the return to initial values; however, at the next administration, the patient again developed grade two liver toxicity. Therefore, a first dose reduction of ribociclib was necessary (ribociclib 200 mg × 2/day given orally). In December 2021, after 4 months of treatment with 400 mg of ribociclib daily, the patient developed grade 3 liver toxicity, with elevations of transaminases 12 times the ULN, so we decided to make a second dose reduction of ribociclib (200 mg/day given orally). During this time, the total bilirubin level was within normal limits. Unfortunately, in February 2022, the patient developed grade 4 liver toxicity with transaminases more than 27 times the ULN, so we had to stop the administration of ribociclib completely. We excluded all other probable causes of hypertransaminasemia, including a possible infectious disease or liver metastases. The patient did not receive any concomitant hepatotoxic medications. Viral serology for hepatitis A, B, and C was also negative. It took a long time (about 5 months) for AST and ALT to return to the initial normal values. The patient received very close monitoring and supportive treatment during this time. Corticosteroid therapy was also necessary after grade four hypertransaminasemia.

The case was discussed again by the multidisciplinary oncological tumor board. It was decided only to continue the treatment with letrozol and osteoclast inhibitors (zoledronic acid 4 mg IV every 4 weeks). Moreover, she was given a daily oral calcium supplement of 500 mg and vitamin D 400 IU, in addition to the zoledronic acid. Therefore, from March to July, the patient was under treatment with AIs and osteoclast inhibitors. During this time, the patient underwent multiple imagistic tests, such as thorax CT and abdominal and pelvis MRI, all of which showed stable oncological disease.

In August 2022, the oncological tumor board decided to initiate treatment with another CDK4/6 inhibitor (abemaciclib 150 mg × 2/day given orally), along with the AI and the osteoclast inhibitor. Therefore, for 6 months, the patient was under treatment with abemaciclib, which was well tolerated until February 2023. At this point, she presented in our clinic with epistaxis and grade three diarrhea. A CBC was performed, and it revealed grade three neutropenia. We decided to temporarily stop the CDK4/6 inhibitor. The diarrhea was treated with hydration, dietary modifications, and loperamide, and the neutropenia decreased to grade one after one week. Therefore, we chose to make a first dose reduction in abemaciclib to 100 mg × 2/day given orally.

**Figure 5:**
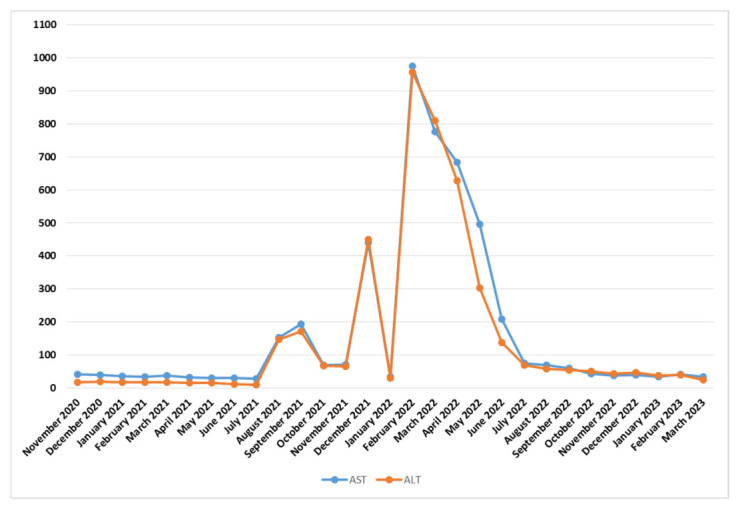
Liver enzyme (AST and ALT) evolution during the treatment.

All the liver enzyme values (collected in dynamics) are graphically illustrated in Figure 5.

**Figure 6:**
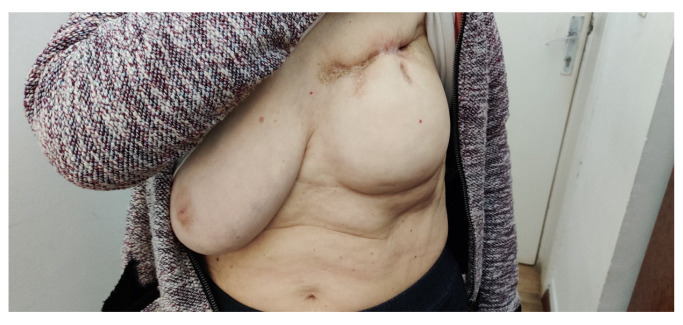
Clinical presentation of the left breast in March 2023.

After the experience of the unusual switch from one CDK4/6 inhibitor to another and the patient’s three types of adverse reactions (liver toxicity from ribociclib and hematological and digestive toxicity from abemaciclib), at the time of writing, we are monitoring her closely to best manage the course of treatment. A significantly improved condition of the left breast (after chemotherapy, radiotherapy, and targeted therapy) can be seen now (Figure 6). Moreover, the CBC, as well as the liver enzymes and biomarkers, are within normal limits. 

All the adverse events were reported to the National Agency of Medicines and Medical Devices in Romania.

## 3. Discussion

Palbociclib, ribociclib, and abemaciclib are well-tolerated, orally active, cyclin-dependent kinase inhibitors. Each of them has been approved for HR+/HER2- metastatic breast cancer following pivotal trials showing substantial PFS benefit in first- and second-line treatments in combination with endocrine therapy. Moreover, abemaciclib has also been approved as a monotherapy for pre-treated patients [3].

From a safety standpoint, CDK4/6 inhibitors are well-tolerated agents with similar safety profiles, although some differences exist in the pattern and frequency of toxicities, which might influence the choice of a given medication [4].

Abemaciclib is structurally distinct from the other two CDK4/6 inhibitors and shows greater selectivity for CDK4 than CDK6 [5], being 14 times more potent against CDK4 than CDK6. CDK4 is more important for breast tumorigenesis, while CDK6 is associated with hematopoietic stem cell differentiation [6]. This is why abemaciclib has a higher toxicity rate of diarrhea and fatigue [7], while ribociclib and palbociclib have a toxicity profile of hematological adverse events, neutropenia being the most common adverse event reported [8,9]. Moreover, ribociclib demonstrates a higher incidence of QT interval prolongation and increased liver enzymes [9].

All three CDK4/6 inhibitors—palbociclib, abemaciclib, and ribociclib—are FDA approved based on pivotal trials: PALOMA-1,2,3 [1,2,3]; MONARCH-1,2,3 [4,5,6]; and MONALEESA-2,7 [7,8], respectively. All of them reported different safety profiles regarding drug-induced toxicity. Below, we summarize the most important highlights of the three toxicities that our patient encountered during treatment, extracted from the pivotal trials of the approved CDK4/6 inhibitors. In addition, we explain the rationale for using ribociclib and abemaciclib.

### 3.1. Rationale for CDK4/6 Inhibitors Choice

Since these three CDK4/6 inhibitors have not been directly compared head-to-head in randomized clinical trials, the choice of the CDK4/6 inhibitor to associate with ET is influenced by differences in safety profile alongside the different schedules of administration and patient comorbidities [9].

In our clinic, we have a wider and richer experience with ribociclib, and we also observed better outcomes in metastatic luminal breast cancer patients treated with this CDK4/6 inhibitor over the others. Moreover, according to the MONALEESA-2 trial, in the endocrine-sensitive setting, a significantly prolonged OS with ribociclib associated with letrozole was observed in post-menopausal women, with a 24% reduction in risk of death and a median survival of 63.9 months [7]. Similarly, in the endocrine-sensitive cohort treated with ribociclib plus fulvestrant as first-line therapy in the MONALEESA 3 trial, the OS benefit was statistically confirmed in the last study update [10]. A similar reduction in death risk was also present in pre- and peri-menopausal patients treated with ribociclib plus ET (AI or tamoxifen) and goserelin in the MONALEESA 7 trial [11].

After the encountered toxicity to ribociclib, it was decided to continue with a CDK4/6 inhibitor given the fact that the patient had multiple metastases and negative prognostic factors, such as a high value of ki67 index, ulcerated breast, and the presence of permeation nodules. We did not expect her to have any other toxicity to the second CDK4/6 inhibitor, and we considered that there was an urgent need to introduce another one, even if the patient had stable oncological disease. Our choice this time was abemaciclib because of its more complex mechanism of action compared to palbociclib (14 times more potent against CDK4 than CDK6). Furthermore, abemaciclib was chosen since the everyday schedule is associated with better patient compliance and a lower risk of dosage mistakes. Moreover, in a meta-analysis conducted by Piezzo et al., ribociclib and abemaciclib confirmed a statistically significant reduction in death risk, while palbociclib was the only class member not showing a statistical hazard ratio per OS [12]. Of note, in the MONARCH 3 trial evaluating abemaciclib plus AI in post-menopausal women with endocrine-sensitive disease, the objective response rate in patients with measurable diseases reached 61%, with a median duration of response of 32.7 months [13].

### 3.2. Elevation of Liver Enzymes

In the PALOMA-3 trial, a grade 1/2 ALT increase was seen in only 4% of the patients, and a grade 3 ALT increase in 3% of the patients in the palbociclib group [10]. In the MONARCH-3 trial, a grade 3 and grade 4 ALT increase was observed in 5.8% and 0.6% of the patients, respectively, and a grade 3 AST increase in 3.8% of the patients. No grade four ALT increase was observed in patients treated with abemaciclib and nonsteroidal AIs [7]. However, in the MONALEESA-2 trial, grade 3/4 ALT and AST elevations occurred in 9.3% and 5.7% of the patients, respectively, following a treatment combination of ribociclib and letrozole [9]. Table 2 summarizes the dose modifications and management of hepatobiliary toxicity of ribociclib.

### 3.3. Gastrointestinal Toxicities

There are three common gastrointestinal side effects associated with CDK4/6 inhibitors: nausea, vomiting, and diarrhea. The appearance rate for these is lower in patients treated with ribociclib and palbociclib. In those treated with abemaciclib, the toxicity profile has a higher rate of grade three diarrhea. In the MONARCH-1 trial, 90% of the patients treated with abemaciclib monotherapy experienced diarrhea, generally within 1 week of treatment initiation, and that led to dose reductions in 21% of the patients. In most cases, it did not last long and was resolved with a median duration of 7.5  days for grade 2 and 4.5 days for grade 3 [11]. In the MONARCH-2 trial, 73% of the patients in the abemaciclib group presented with grade 1 or 2 diarrhea and 13.4% with grade 3 diarrhea. It typically occurred in the first treatment cycle and was effectively managed using antidiarrheal medications and dose adjustments [12]. In the MONARCH-3 trial, 27.2% of the patients had grade 2 diarrhea, only 9.5% of patients had grade 3 diarrhea, and none developed grade 4 diarrhea [7].

An exploratory analysis was performed to examine the potential relationship between early toxicities associated with abemaciclib and the PFS of patients. Compared with the placebo group, patients treated with abemaciclib who had diarrhea within the first 7 days or who did not have diarrhea within the first 7 days showed an improvement in PFS. A time-dependent analysis was performed to examine the association between dose level (150, 100, and 50 mg) and PFS. Compared with the PFS of patients treated at the 150 mg dose level, there was no apparent difference in PFS for patients reduced to 100 mg or 50 mg [13].

Table 3 summarizes the dose modifications and management of diarrhea caused by abemaciclib. 

### 3.4. Neutropenia

In contrast to neutropenia caused by chemotherapy, CDK4/6 inhibitor-associated neutropenia is rapidly reversible and considerably less frequent. This is because CDK4/6 inhibitors induce cell-cycle arrest by decreasing the proliferation of hematopoietic stem cells and allow the cell cycle to resume following a reduction or interruption in dose [14]. After using an in vitro assay with human bone marrow mononuclear cells, the investigators showed that palbociclib-induced bone marrow suppression occurred through cell-cycle arrest without DNA damage and apoptotic cell death that is usually seen with cytotoxic chemotherapies. Furthermore, neutropenia often decreases with each cycle, suggesting that cumulative toxicity does not exist [14].

However, neutropenia is the most common grade 3/4 adverse event observed in all clinical trials. Abemaciclib has a greater CDK4 selectivity, so it has a 50% lower neutropenia rate (in all grades) than palbociclib and ribociclib [13]. In the MONARCH-3 trial, 41.3% of the patients in the abemaciclib group experienced neutropenia, of which 16.2% had neutropenia grade 2, 19.6% had grade 3, and 1.5% had grade 4. All grades of neutropenia were generally observed by cycle two, and grade three and four neutropenia were uncommonly observed during the later cycles [15]. In the MONALEESA-2 trial, neutropenia occurred in 63.8% of the patients in the ribociclib group [16]. In the PALOMA trial, grade 3 or 4 neutropenia occurred in 70% of the patients receiving palbociclib–fulvestrant, with febrile neutropenia remaining uncommon, occurring only in 1% of the patients [8].

In Table 4, we summarize the dose modifications and management of neutropenia caused by abemaciclib.

In Table 5, we summarize these three adverse events and the occurrence percentage in the three pivotal trials for CDK4/6 inhibitors approval.

Regarding the overall survival (OS), our patient has had an OS of 16 months to date. We consider this to be a very good survival rate for this patient, given the limiting toxicities she encountered, and the visceral crisis that she had early in her treatment, for which we had to initiate chemotherapy.

### 3.5. Similar Cases of Rechallenge in the Literature

There are a few cases described in the specialty literature regarding rechallenge to CDK4/6 inhibitors. Meynard et al. [17] reported a case in France of palbociclib rechallenge after hepatic toxicity to ribociclib. The patient encountered grade 3 hypertransaminasemia after 16 weeks of ribociclib full dose (600 mg/day). The patient did not have any history of hepatic disease or liver metastases. Increased transaminases lasted for 14 weeks. Palbociclib was introduced at 75 mg/day and increased to 100 mg/day. After one year, the patient remained free of any toxicities. A series of case presentations found in the literature was reported by Fuentes-Antras and colleagues [3] from Spain. They reviewed 6 patients in whom ribociclib was discontinued due to grade ≥3 hypertransaminasemia, and who subsequently received palbociclib or abemaciclib. The median number of cycles of ribociclib was 4.5 before treatment discontinuation. None of them had a history of previous liver disease. The median time until the values normalized was 13.5 weeks. The drugs of choice for the rechallenge strategy were palbociclib plus fulvestrant in four patients, palbociclib plus letrozole in one patient, and abemaciclib plus letrozole in one patient. The median follow-up after treatment discontinuation was 19.5 months, and none of the 6 patients encountered another toxicity. Another case series is from Zagreb, reported by Jelena et al. [18], in which three patients switched from ribociclib to palbociclib and fulvestrant, and one to abemaciclib and fulvestrant after prior grade three and four hypertransaminasemia due to ribociclib. No hepatotoxicity was observed after the drug switch.

## 4. Conclusions

To the best of our knowledge, we have presented the only case described in the literature of metastatic breast cancer with toxicity developed in reaction to a CDK4/6 inhibitor and two toxicities in reaction to another CDK4/6 inhibitor after rechallenging treatment due to former toxicity. We reported the case of a patient with three different toxicities (grade four liver toxicity, grade three diarrhea, and grade three neutropenia) to two different CDK4/6 inhibitors (ribociclib and abemaciclib). However, we succeeded in managing all of them with supportive treatment and close monitoring of the patient. Currently, we continue to monitor her to see if she remains free of any clinical or biological toxicities.

We believe that this case shows that it is feasible to rechallenge a CDK 4/6 inhibitor by switching to a different drug from the three available. However, the reintroduction of a CDK 4/6 inhibitor should be accompanied by close biological monitoring to avoid the reappearance of hepatic injury or other adverse reactions. To date, the scarcity of data on cross toxicity and cross efficacy between CDK4/6 inhibitors limits the use of rechallenge strategies in patients who need to discontinue one of them because of toxicity. However, we recommend large, randomized trials be condthreeucted in the future.

## Figures and Tables

**Table 1:** Blood tests before targeted therapy initiation.

Parameter	Result	Normal Range
Hemoglobin	13.8	11.7–15.9/g/dL
Hematocrit	5.30	35.0–47.0/%
Platelets	342	150–400/× 10^2^/uL
White blood cells	5.49	4.6–10.2/× 10^9^/uL
Neutrophils	3.36	1.5–6.9/× 10^3^/uL
Lymphocytes	1.56	0.6–3.4/× 10^3^/uL
Monocytes	0.47	0–0.9/× 10^3^/uL
Eosinophils	0.06	0–0.7/× 10^3^/uL
AST	41	14–36/U/L
ALT	18	5–35/U/L
eGFR CKD-EPI	89	>90/L/min/1.73 m^2^
Total Bilirubin	0.8	0.2–1.3/mg/dL
Urea	49	15–50/mg/dL
Creatinine	0.7	0.7–1.2/mg/dL
Sodium	142	137–145/mmol/L
Potassium	4.9	3.6–5.1/mmol/L
LDH	439	120–246/U/L
Glucose	104	65–105/mg/dL
Erythrocyte sedimentation rate	26	0–20/mm/1 h
Calcium	1.91	2.0–2.5/mEg/L
Triglycerides	153	35–150/mg/dL

**Table 2:** Dose modifications and management of hepatobiliary toxicity of ribociclib, according to ribociclib US prescribing information, accessed in March 2023.

	Grade 1 (>3 × ULN)	Grade 2 (>3 to 5 × ULN)	Grade 3 (>5 to 20 × ULN)	Grade 4 (>20 × ULN)
AST and/or ALT elevations from baseline **, without increase in total bilirubin above 2 × ULN	No dose adjustment is required.	Baseline grade <2: Dose interruption until recovery to ≤ baseline grade, then resumption of Kisqali at same dose level. If grade 2 recurs, resume Kisqali at next lower dose level. Baseline grade = 2: No dose interruption	Dose interruption of Kisqali until recovery to ≤ baseline grade, then resumption at next lower dose level. If grade 3 recurs, discontinue Kisqali.	Discontinue Kisqali.
Combined elevations in AST and/or ALT together with total bilirubin increase in the absence of cholestasis	If patients develop ALT and/or AST >3 × ULN along with total bilirubin >2 × ULN irrespective of baseline grade, discontinue Kisqali.			

** Baseline = prior to treatment initiation. ULN = upper limit of normal.

**Table 3:** Dose modifications and management of diarrhea caused by abemaciclib, according to abemaciclib US prescribing information, accessed in March 2023.

Toxicity	Management Recommendations
Grade 1	No dose adjustment required.
Grade 2	If toxicity does not resolve within 24 h to Grade 1 or less, suspend dose until resolution. Dose reduction is not required.
Grade 2 that persists or recurs after resuming the same dose despite maximal supportive measures	Suspend dose until toxicity resolves to Grade 1 or less. Resume at next lower dose.
Grade 3 or 4 or requires hospitalization	

**Table 4:** Dose modifications and management of neutropenia caused by abemaciclib, according to abemaciclib US prescribing information, accessed in March 2023.

Toxicity	Management Recommendations
Grade 1 or 2	No dose adjustment required.
Grade 3	Suspend dose until toxicity resolves to Grade 2 or less. Dose reduction is not required.
Grade 3, recurrent; or Grade 4	Suspend dose until toxicity resolves to Grade 2 or less. Resume at next lower dose.
Patient requires administration of blood cell growth factors	Suspend abemaciclib dose for at least 48 h after the last dose of blood cell growth factors was administered and until toxicity resolves to Grade 2 or less. Resume at next lower dose unless the dose was already reduced for the toxicity that led to the use of the growth factor.

**Table 5:** Liver toxicity, diarrhea, and neutropenia occurrence in the three pivotal trials for CDK4/6 inhibitors.

	AST		ALT		Diarrhea		Neutropenia	
	Grade 1 + 2 %	Grade 3 + 4 %	Grade 1 + 2 %	Grade 3 + 4 %	Grade 1 + 2 %	Grade 3 + 4 %	Grade 1 + 2 %	Grade 3 + 4 %
MONALEESA-2 (N = 334)	44	7	46	10	33.8	1.2	33	60
MONALEESA-7 (N = 248)	37	4.8	33	6	NA	NA	29	63
MONARCH-2 (N = 441)	33.1	3.9	36.4	4.6	73	13	54.5	32.5
MONARCH-3 (N = 327)	33.2	3.8	41.4	6.6	72	9	58.1	21.9
*PALOMA-2* *(N = 444)*	*49*	*3*	*40*	*3*	*25*	*1*	*14*	*66*
*PALOMA-3* *(N = 345)*	*39*	*4*	*34*	*2*	*24*	*0*	*17*	*66*

## Data Availability

No new data was created.

## Abbreviations

ULN	Upper Limit of Normal
AI	Aromatase Inhibitor
HR	Hormone Receptor
HER2	Human Epidermal Growth Factor 2
Er	Estrogen receptor
PgR	Progesterone receptor
Ki67	Nuclear antigen that is an excellent marker of active cell proliferation
PET-CT	Positron Emission Tomography
SUV	Standardized Uptake Values
M1HEP	Hepatic metastases
M1LYM	Lymphatic metastases
M1ADR	Adrenal metastases
G-CSF	Granulocyte Colony-Stimulating Factor
NCCN	National Comprehensive Cancer Network
IV	Intravenous
EKG	Electrocardiography
CBC	Complete Blood Count
PTV50	Median Planning Target Volume
Gy	Gray
Fx	Fractions
IMRT-VMAT	Intensity Modulated Radiotherapy—Volumetric Modulated Arc Therapy
ALT	Alanine Aminotransferase
AST	Aspartate Aminotransferase
CT	Computed Tomography
PFS	Progression Free Survival
OS	Overall Survival
